# Development and Validation of the Unesp-Botucatu Goat Acute Pain Scale

**DOI:** 10.3390/ani13132136

**Published:** 2023-06-28

**Authors:** Mariana Werneck Fonseca, Pedro Henrique Esteves Trindade, Renata Haddad Pinho, André Augusto Justo, Rubia Mitalli Tomacheuski, Nuno Emanuel de Oliveira Figueiredo da Silva, Heraldo Cesar Gonçalves, Stelio Pacca Loureiro Luna

**Affiliations:** 1 Department of Surgical Specialties and Anesthesiology, Botucatu Medical School, São Paulo State University (Unesp), Botucatu 18618-687, SP, Brazil; mariana.werneck@unesp.br (M.W.F.); or pesteve@ncsu.edu (P.H.E.T.); renata.pinho@ucalgary.ca (R.H.P.); or rmtomach@ncsu.edu (R.M.T.); emmanuno@gmail.com (N.E.d.O.F.d.S.); 2 Department of Population Health and Pathobiology, College of Veterinary Medicine, North Carolina State University (NCSU), Raleigh, NC 27695, USA; 3 Faculty of Veterinary Medicine, University of Calgary, 3280 Hospital Dr NW, Calgary, AB T2N 4Z6, Canada; 4 Department of Veterinary Medicine, School of Animal Science and Food Engineering, University of São Paulo (USP), Pirassununga 13635-900, SP, Brazil; andre.justo@usp.br; 5 Department of Animal Production and Preventive Veterinary Medicine, School of Veterinary Medicine and Animal Science, São Paulo State University (Unesp), Botucatu 18610-034, SP, Brazil; heraldo.cesar@unesp.br; 6 Department of Veterinary Surgery and Animal Reproduction, São Paulo State University (Unesp), Botucatu 18618-681, SP, Brazil

**Keywords:** animal welfare, caprine, pain, pain measurement, pain scale, postoperative care, reliability, validation study

## Abstract

**Simple Summary:**

In livestock animals, such as goats, many routine management procedures in production systems, e.g., castration and dehorning, are painful, and most are performed without proper anesthetic or analgesic protocols for pain management. Validated pain assessment scales are important to detect pain and indicate painkillers to improve the welfare of these animals. Goats are one of the few production animal species that does not have a pain assessment scale available in the literature. We aimed to develop and validate the Unesp-Botucatu goat acute pain scale (UGAPS). After video observations of goats before and after castration, the scale was developed based on a list of behaviors and experts’ opinions. The videos were watched by four observers unaware of the time point when the videos were recorded. After the scale was statistically refined, UGAPS showed very good (≥86%) agreement between the same evaluator assessment of pain scores observed at the same videos assessed in different time points and very good agreement (≥85%) between evaluators’ assessments, adequate interaction between its items, 99% precision to detect an absence of pain before surgery, 90% precision to detect pain after surgery, and an indicative score ≥3 of a total of 10 points suggestive of analgesia in goats submitted to castration.

**Abstract:**

We aimed to develop and validate the Unesp-Botucatu goat acute pain scale (UGAPS). Thirty goats (5 negative controls and 25 submitted to orchiectomy) were filmed for 7 min at the time points 24 h before and 2 h, 3 h (1 h after analgesia), and 24 h after orchiectomy. After content validation, according to an ethogram and literature, four blind observers analyzed the videos randomly to score the UGAPS, repeating the same assessment in 30 days. According to the confirmatory factor analysis, the UGAPS is unidimensional. Intra- and interobserver reliability was very good for all raters (Intraclass correlation coefficient ≥85%). Spearman’s correlation between UGAPS versus VAS was 0.85 confirming the criterion validity. Internal consistency was 0.60 for Cronbach’s α Cronbach and 0.67 for McDonald’s ω. The item-total correlation was acceptable for 80% of the items (0.3–0.7). Specificity and sensitivity based on the cut-off point were 99% and 90%, respectively. The scale was responsive and demonstrated construct validity shown by the increase and decrease of scores after surgery pain and analgesia, respectively. The cut-off point for rescue analgesia is ≥3 of 10, with an area under the curve of 95.27%. The UGAPS presents content, criterion, and construct validities, responsiveness, and reliability to assess postoperative pain in castrated goats.

## 1. Introduction

Identifying and preventing pain in production animals is one of the steps to improve animal welfare. One of the “Five Freedoms” governing animal welfare is being “free from pain, injury, and disease, through prevention or prompt diagnosis and treatment” [1]. Production animals, such as goats, still suffer from routine practices and procedures to facilitate handling, such as castration, hot iron and cold branding, and dehorning, however, these practices result in pain and compromise animal welfare [2,3,4].

Until recently, the difficulty in recognizing pain in production animals was due to the lack of reliable validated instruments for its assessment, which contributed to oligoanalgesia in certain species [5,6,7]. This deficiency has been overcome in some species of production animals, such as cattle [8], swine [9], and sheep [10], but goats are one of the few species of domestic and production animals for which there is not yet a validated species-specific scale.

Behavior seems to be the best indicator for assessing pain, given the lack of verbal expression in animals, as it is easy to observe, does not require equipment or animal restraint, does not generate stress, and is cost-effective, being applicable in clinical and experimental studies [11,12]. Although a validated pain scale for sheep is already available [10], the expression of pain between goats and sheep differs. Different from sheep, vocalization is more frequent in goats with pain [13]. Goats subjected to dehorning vocalize and kick the ground [2]. Unlike lambs, castrated kids tend to hide in the mother’s absence [13].

For pain scales to be reliable, accurate, and species-specific, they must be assessed using validity and reliability criteria already established in other animal species [14,15,16,17,18,19,20,21,22,23,24,25], and that follow international validation guidelines. Considering the lack of validated tools to assess pain in goats, the present study aimed to develop and validate a scale to assess acute pain in goats using a methodology similar to that used in other species of domestic animals and following the guidelines from COSMIN [7,24,25].

## 2. Materials and Methods

The study was approved by the Animal Ethics and Experimentation Committee of the Faculty of Veterinary Medicine and Animal Science (FMVZ), UNESP-Botucatu, under protocol number 0327/2023.

### 2.1. Animals and Experimental Design

Thirty male goats from the FMVZ-Unesp farm with a mean age of 6 ± 1 (5–7) months and a mean weight of 17.5 (10.5–25.2) kg were used (*Capra aegagrus hircus*). Their breeds were Anglo-Nubian (*n* = 22) and Alpine-Brown (*n* = 8). Five of these animals served as controls (two Alpine-Browns and three Anglo-Nubians) and did not undergo orchiectomy. As inclusion criteria, healthy goats were selected based on physical examination (body score assessment, cardiac and pulmonary auscultation, rectal temperature, and palpation of lymph nodes) and laboratory examination (hematocrit and plasma protein).

The goat housing had 10 bays with ventilation and sunlight controlled by metal windows. All animals were well adapted to the facilities because they had lived in the same accommodation since birth. The pens contained a tire for environmental enrichment. The animals were kept in the same group in which they were previously allocated in each 12 m^2^ wooden pen (4 × 3 m) (Figure S1A). During the study period the daily temperature varied between 16 and 24 °C and relative air humidity was between 57.3–80.3%. Identification of the coat and the individual morphology were recorded in photographs (Figure S1B).

### 2.2. Surgery and Anesthesia

Elective orchiectomy (open technique) was performed in twenty-five goats between 8 am and 10 am in a single day. The orchiectomy was carried out after physical restraint, antisepsis with 2% chlorhexidine detergent followed by 0.5% alcoholic chlorhexidine, infiltrative blockade in the incision line (scrotal raphe) with 1 mL of 2% lidocaine (2% Xylestesin, Cristália Produtos Farmacêuticos LTDA, São Paulo, SP, Brazil) without vasoconstrictor, and intratesticular anesthesia with 1 mL of local anesthetic in each testicle. After five minutes of local blockade, the same experienced surgeon performed an orchiectomy on all the animals, after which each animal was taken to its original stall.

### 2.3. Filming

**Figure 1:**
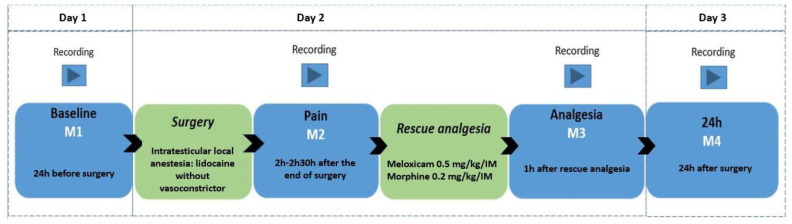
Study timeline flowchart with time points and filming time points, local anesthesia, and analgesia protocol.

A camera (GoPro HERO5 Black, GoPro Inc., San Mateo, CA, USA) positioned in the left corner of the bay was remotely activated by the researcher, M.W.F., at a minimum distance of 4 m. The animals were filmed for seven minutes without the presence of an observer to use the recordings in another parallel study. The observer then approached the stall and always positioned herself in the same place, activating the filming by remote control for another seven minutes. For filming, the goats were divided into 10 video-recording groups (pens) containing 4 goats each, including 1 or 2 control goats (not submitted to an orchiectomy—without pain). The goats were filmed in four periods: 24 h before the orchiectomy (8:00 am–10:00 am; *baseline*—M1), between 2 and 2.30 h after the end of the orchiectomy (12:00–2:30 pm; *pain*—M2), 1 h after the *postoperative analgesia* (M3) performed with 0.2 mg/kg of morphine (Dimorf 1%, Cristália Produtos Farmacêuticos LTDA, São Paulo, SP, Brazil), and 0.5 mg/kg meloxicam (Maxicam 2%, Ourofino, São Paulo, SP, Brazil) intramuscularly (IM) in all goats, and 24 h after the end of the orchiectomy (M4) (Figure 1).

### 2.4. Ethogram and Selection of Behaviors

The main researcher (M.W.F.) elaborated an ethogram using the seven-minute footage of the preoperative periods with the presence of the observer (5 control animals and 25 animals submitted to an orchiectomy) in all time points (30 animals × 4 time points = 120 videos). Through the focal animal method [26], each video was evaluated to identify the occurrence of all behaviors in each individual. After the analysis, 40 behaviors were listed and described (Appendix A). Then, the videos were analyzed again, as many times as necessary to measure the frequency and duration of the selected behaviors.

### 2.5. Creation of the Pain Scale (UGAPS)

According to the ethogram, all behaviors indicative of pain that presented a higher frequency of occurrence or duration in the time point of *pain* (M2) compared to the *baseline* (M1) or that presented a reduction in frequency or duration after *analgesia* (M3) in relation to *pain* (M2) were included in the scale. The opposite was adopted for maintenance behaviors (normal). The same analysis was performed to assess the effect of the time of day on the behavior of negative control animals. Behaviors were grouped into seven items and separated according to maintenance (normal; score 0) or pain (score 1) (Appendix B).

### 2.6. Assessment of the Pain Scale (UGAPS)

The final three minutes of all videos were entirely excluded to minimize evaluator fatigue. No further editing was performed on the videos. The 100 videos of animals submitted to an orchiectomy were randomized according to the time point of filming and animals (www.randomizer.org) to blind the evaluators.

Two female (R.H.P. and R.M.T.) and two male (N.E.O.F.S. and A.A.J.) veterinarians with previous experience in animal pain scale assessments [10,23] watched the videos. Training was provided before the beginning of the evaluations, consisting of an online presentation of demonstrative videos of each behavior (Table 1) and instructions for completing the scale. Afterward, the evaluators carried out the first evaluations within four weeks (25 videos/week). After a four-week break, the same 100 videos were randomized and evaluated again within another four weeks.

The evaluators were instructed to watch the videos as many times as they deemed necessary to record the behaviors and not to exceed 1 h of evaluation per day to avoid fatigue. After watching each video, they recorded and answered the following assessments in order: (1) Would you indicate analgesia for this animal? (yes or no); (2) Visual Analog Scale (VAS)—0 cm without pain and 10 cm the worst possible pain; (3) Proposed pain scale, and (4) How many times did you watch each video?

### 2.7. Statistical Analysis

Statistical analyses were conducted by a data scientist (PHET) in R software with the RStudio integrated development environment (Version 4.1.0; 29 June 2021; RStudio, Inc., Boston, MA, USA). The functions and packages used were presented in the format “package::function” corresponding to the computational programming language in R. For all tests, a significance level of 5% was considered. All figures were constructed with a palette of colors distinguishable by colorblind people (ggplot2::scale_colour_viridis_d).

The pattern of behavioral occurrence over the periods of the day, percentage of occurrence of each UGAPS item in the peri-operative period, intra- and interobserver reliability [23,27,28,29], multiple association and dimensional structure [23,30,31], internal consistency [32,33,34,35], item-total correlation [34], specificity [34], sensitivity [34], criterion validity [36], importance (weight) of each UGAPS item for pain diagnosis [34,37,38], responsiveness [10], construct validity [23], optimal cut-off point indicating analgesia, and diagnostic uncertainty zone [39,40,41,42] were analyzed as described in detail before (Table 3 of reference [23]).

To select the best set of behavioral items to compose the final version of the UGAPS, a refinement was conducted based on statistical analyses. The remaining items then made up the final version of the UGAPS and were submitted to the validation process.

## 3. Results

### 3.1. Surgery and Anesthesia

The orchiectomy lasted an average of 5 ± 2 min, and there were no trans- or postoperative complications.

### 3.2. Evaluation of the Videos

The medians (minimum and maximum) of the number of times each observer viewed the videos in phase 1 for evaluators 1, 2, 3, and 4 were, respectively, 2(1–3), 1(1–3), 2(1–4), and 1(1–3). In the second evaluation phase, the results were similar, 2(1–3), 1(1–3), 1(1–3), and 1(1–3).

### 3.3. Refinement of the UGAPS

Of the 40 behaviors cataloged for the ethogram, 21 were relevant to assess pain, 10 of which were maintenance behavior (normal) and 15 were characteristic of pain with a lower or higher frequency of occurrence or duration at M2 than M1, respectively. These behaviors comprise the scale assessed by the evaluators (Appendix B). After the evaluation of the scale, 10 statistical analyses were performed to refine and confirm content validation of the UGAPS items before inclusion or not in the definitive scale. The behaviors approved in ≥6 items of a total of 10 were included in the final version of the UGAPS (frequency of occurrence, intra- and interobserver reliability, responsiveness, expert opinion, specificity, sensitivity, item-total correlation, principal component analysis, internal consistency, and occurrence over the day). The final version of the UGAPS comprised five main items and ten subitems, which were assessed for their presence or absence (Table 1).

### 3.4. Pattern of Behavioral Occurrence over the Periods of the Day

Some behaviors evaluated in the ethogram, such as “locomotion—walks or runs”, “appetite—present”, “attitude—vocalizes”, “interaction—interacts with the environment”, and “interaction—headbutt”, were influenced by the time of day in the control group and showed greater frequency or duration in the morning period (M1 and M4). However, after refinement of the scale, the only behaviors that showed influence over the periods of the day and remained on the scale were “locomotion—walks or runs” and “attitude—vocalizes”.

### 3.5. Frequency of Occurrence

**Figure 2:**
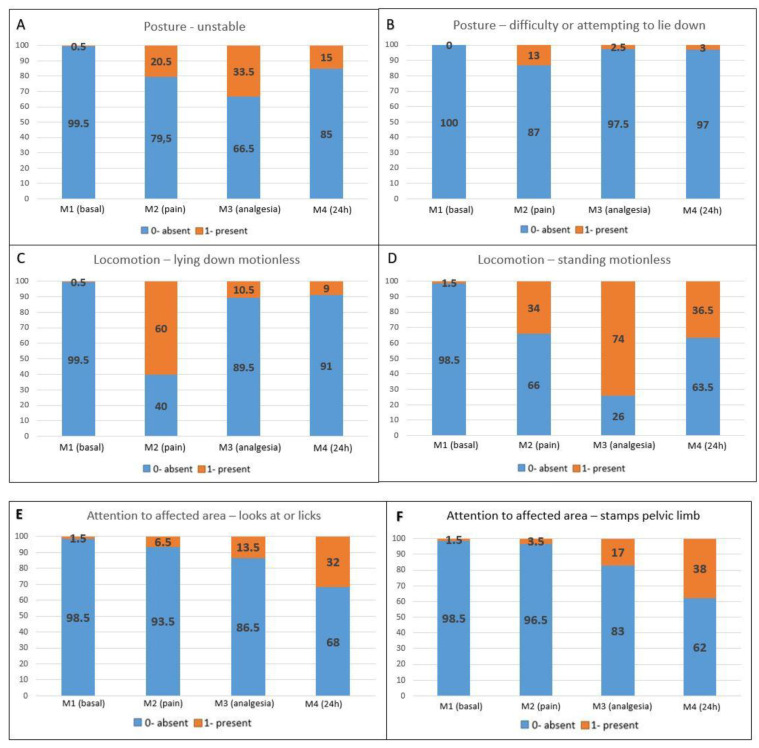

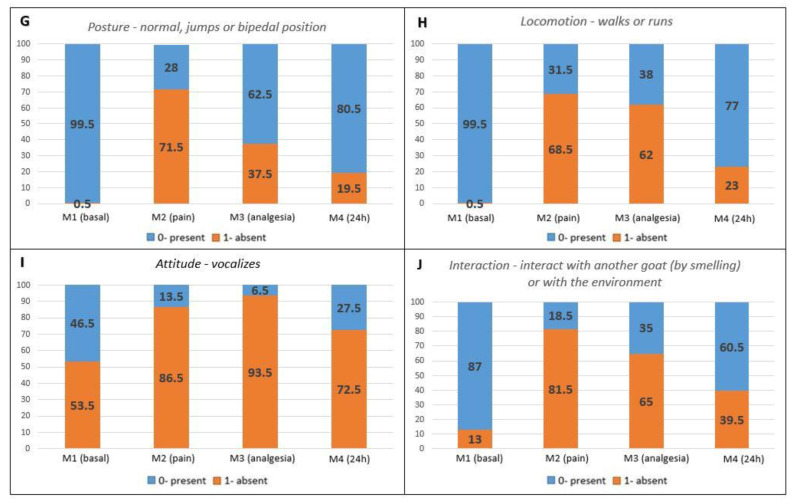
Percentage distribution of scores for each item on the UGAPS in the perioperative period. (**A**–**F**) Pain-related behaviors in which 0—absent and 1—present; (**G**–**J**) Maintenance behaviors where 0—present and 1—absent.

Pain-related behaviors presented a higher occurrence in the postoperative periods compared to M1 (*baseline*) (Figure 2A–F). Maintenance behaviors not related to pain were more markedly reduced at M2 (*pain*), followed by M3 (*analgesia*) and M4 (*24* h) when compared to M1 (*baselin*e) (Figure 2G–J).

### 3.6. Intraobserver Reliability

The four raters achieved very good (>0.81) or good (0.61–0.80) intraobserver reliability (repeatability) on most UGAPS items. Repeatability for the total UGAPS score was very good (≥0.86).

### 3.7. Interobserver Reliability

Interobserver reliability (reproducibility) ranged from poor (<0.2) to very good (0.08–0.92), being moderate (0.41–0.60), good (0.61–0.80), or very good (0.81–1) for most items. Reproducibility for the total UGAPS score was very good (≥0.85) among all interobserver comparisons.

### 3.8. Multiple Association and Dimensional Structure

Horn’s parallel analysis indicated the retention of the first five factors out of a total of nine generated by the exploratory factor analysis. All UGAPS items showed loading values ≥0.50 or ≤−0.50 [31] in at least one factor, except “attention to affected area: looks at or licks”. The five-dimensional structure was submitted to confirmatory factor analysis; however, it was not possible to estimate the chi-square parameters, comparative fit index, Tucker–Lewis index, mean square root of approximation error, or Akaike and Bayesian information criteria, which suggests a unidimensional structure.

### 3.9. Internal Consistency and Item-Total Correlation

The internal consistency values given by Cronbach’s alpha (α) and McDonald’s omega (ω) coefficients between the UGAPS items were close to the minimally acceptable limit (α of 0.60 and ω of 0.67) (Table 2). Most items (α) and half of the items (ω) were relevant to the set, decreasing their α or ω when excluded. The item-total correlation ranged from −0.12 to 0.68. Eight out of ten had an accepted correlation coefficient between 0.3 and 0.7 (Table 2).

The correlogram with heat map illustrates the correlations between the UGAPS items (Figure S2).

### 3.10. Specificity and Sensitivity

All items presented moderate to good specificity (70–99%), except for “attitude—vocalizes” (62%). Half of the items were sensitive (>70%). The other items showed sensitivity between 51% and 69% (Table 3). The specificity and sensitivity of the whole scale were excellent (99%) and good (90%), respectively.

### 3.11. Criterion Validity

The high correlation of the sum of the UGAPS with the VAS according to Spearman rank correlation coefficient (0.85; 0.83–0.87) (*p* < 0.0001) [36] and the agreement between the observers (reproducibility) confirmed concurrent criterion validity. Predictive criterion validity was confirmed by the results of sensitivity which showed that 90% of goats should receive rescue analgesia according to the Youden index in the time point of greatest pain (pain) (Table 3—sensitivity).

### 3.12. Weightings

Wald’s statistic highlighted “posture—normal, jumps, or bipedal position”, “interaction”, “locomotion—standing still”, “attention to affected area—looks at or licks” and “stamps pelvic limb” as the five most important UGAPS behavioral items according to the multilevel binomial logistic regression model (Figure S3).

### 3.13. Responsiveness

**Figure 3:**
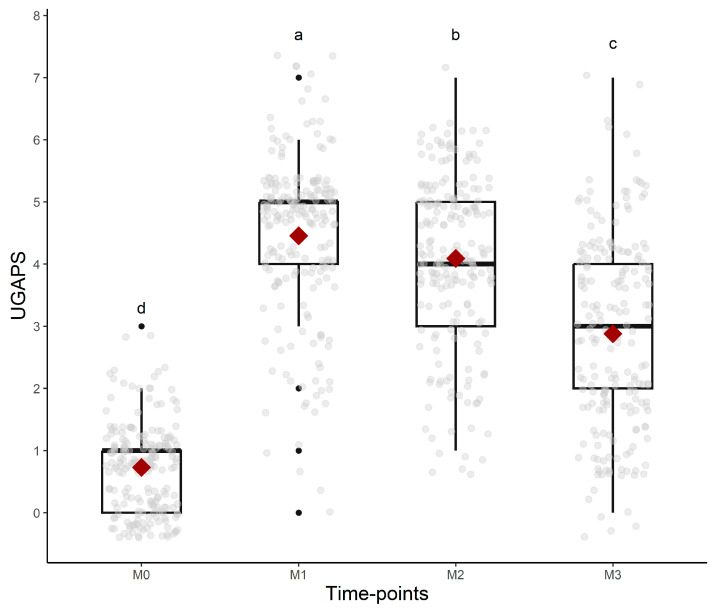
Boxplots of the scores (median/amplitude) of the UGAPS over time. The top and bottom box lines represent the interquartile range (25 to 75%); the line within the box represents the median; the extremes of the whiskers represent the minimum and maximum values; black lozenges (♦) represent the mean, and black circles (●) represent outliers. Different letters express significant differences between time points where a > b > c > d.

All UGAPS items except “attention to affected area—looks at or licks” and “stamps pelvic limb” were responsive to pain because scores were higher after surgery compared to baseline, but only the scores of “posture: normal, jumps, or bipedal”, “posture: difficulty lying down”, “locomotion: lying down motionless”, and “interaction” reduced after rescue analgesia. Both behaviors related to “attention to affected area” peaked at 24 h. For the indication of rescue analgesia, VAS, and the sum of the UGAPS, the hypothesis was confirmed that scores were higher at M2 (post-orchiectomy), followed by M3 (post-analgesia), M4 (24 h post-orchiectomy), and M1 (pre-orchiectomy) (Table 4, Figure 3). The evaluators (as fixed effects) influenced the scores of UGAPS. The scores of evaluator 2 were higher than evaluator 1 (*p* = 0.02) and 3 (*p* = 0.009). There were no effects of phase (*p* = 0.98) and breed (*p* = 0.31).

### 3.14. ROC Curve, Cut-Off Point, and Diagnostic Uncertainty Zone

**Figure 4:**
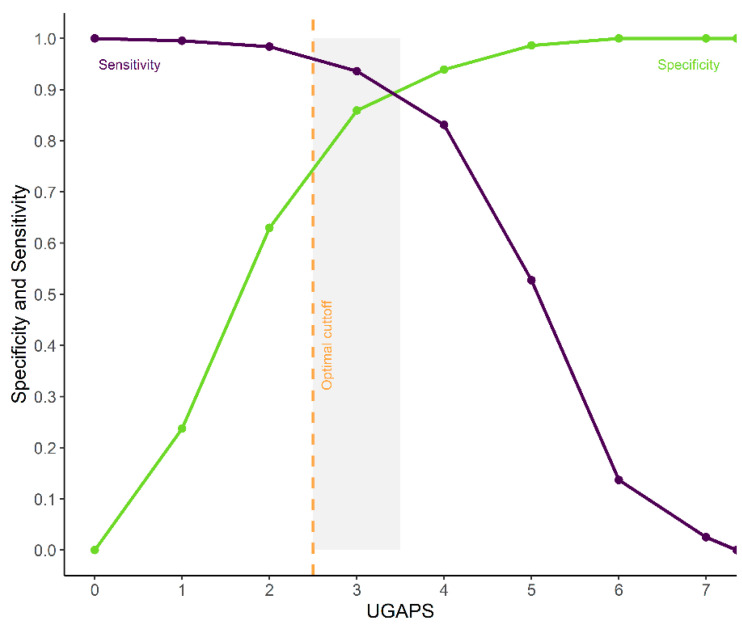
Receiver operating characteristic (ROC) curve of two graphs with the diagnostic uncertainty zone for the UGAPS. The diagnostic uncertainty zone scores ranged from 2.5 to 3.5; therefore, scores ≤2 indicate truly negatives (goat without pain) and ≥4 indicate truly positives (goat suffering pain). A Youden index ≥3 (>2.5) represents the cut-off point for indication of rescue analgesia.

According to the ROC curve, the UGAPS cut-off point for rescue analgesia was determined at 2.5 (≥3) to differentiate goats with pain from those without pain (Figure 4, Table 5). The 95% confidence intervals replicating the original ROC curve 1.001 times by the bootstrap method were between 94 and 97%. The diagnostic uncertainty zone is between 2.5 and 3.5, therefore, scores ≤ 2 indicate true negatives (goat without pain) and ≥4 indicate true positives (goat suffering pain) (Figure 4).

The AUC was 95.27%, which demonstrates the excellent discriminatory ability of the UGAPS. The optimal cut-off point was maintained when excluding “vocalizes” or “walks or runs” separately but was reduced to 1.5 (≥2) if both behaviors were removed. The cut-off point determined by the Youden index was ≥3.35 for VAS (Table 5).

The percentage of scores within the diagnostic uncertainty zone varied between 24% (at M2) and 50% (at M4).

## 4. Discussion

This study is pioneering in developing and testing the psychometric properties of a scale to assess acute postoperative pain in goats submitted to orchiectomy, since, to date, there is no validated pain scale for this species [7]. The results contribute to the detection and quantification of pain and facilitate decision-making for analgesic intervention in goats. It is expected that the identification of pain will benefit the animal welfare of the goat species [2,43]. The UGAPS presented content, criterion, and construct validity, reliability, and responsiveness to recognize pain after orchiectomy, as well as generated a cut-off point suggesting the need for analgesia.

For validation of the scale’s content, an ethogram created before the development of the UGAPS identified the relevant behaviors that changed after the surgery. Through the ethogram, characteristic pain behaviors can be differentiated from usual behaviors, characterized as maintenance ones. Behaviors expressing pain increased in frequency or duration after surgery and the opposite occurred for maintenance behaviors. According to the approval criteria used for the analysis of frequency or duration of occurrence greater than 10% among all time points versus baseline, the maintenance behaviors “attitude—digging” and “attitude—self-cleaning” were excluded, as well as “interaction with the environment (tire)” due to their low occurrence. Other behaviors, in addition to low occurrence, appear to be characteristic of the sample group of male animals, such as “mounting”, “Flehmen response”, “headbutting”, and “smelling genitalia”, therefore, they were not included in the scale. Thus, the four approved maintenance behaviors that made up the scale, “posture—normal, jumps, or bipedal position”, “locomotion—walks or runs”, “attitude—vocalizes”, and “interaction” seem to be important to suggest, when absent, the occurrence of pain in this species, as some of them in other species [8,9,10,15,16,17,18,21,22,23], since they reduced after the surgery (*pain*).

Thus, using this frequency or duration of occurrence criterion, less relevant behaviors were excluded, reducing the time required to evaluate the scale. Behaviors related to pain in goats may vary according to age and type of procedure [44,45,46], and in the method of castration used [44]. Goats of exotic breeds, such as Anglo-Nubian and Alpine-Brown, originating in England and Switzerland, respectively, reach maturity at 6 to 8 months of age and/or when they have completed body development. Animals of exotic breeds bred in tropical areas, such as Brazil, tend to reach sexual maturity later [47]. In this way, when considering the age, weight, and region of our study, it can be considered that the animals had not reached maturity and could be considered young.

Some behaviors related to the presence of pain identified in this study had already been described in goats and other species, such as “tremor” and “arching the back”, but were excluded after refinement of the scale [23,48]. Approved pain behaviors such as “standing still” and “lying down motionless” have already been described for ruminants. The locomotion pattern was also affected in sheep after a laparoscopy [10], in goat kids after castration [44], and in goat kids after cautery disbudding [45,46].

Like in rabbits [49] and horses [50], the expression of pain can be inhibited with the presence of an observer, increasing the possibility of false negative results (animals in pain that do not express pain). However, we chose to evaluate the videos with an observer present because this would be the most frequent situation for evaluating pain in field situations on farms when remote viewing by cameras is not available. A future study can either confirm or not the influence of the observer’s presence in caprine pain assessment.

Another factor that interferes with behavioral expression is the time of day, as reported in horses [51]. It was observed that two maintenance behaviors (“locomotion—walks or runs” and “attitude—vocalizes”) showed differences in the pattern of occurrence over the periods of the day. Vocalization is a characteristic behavior of goats and is reported more in this species than in sheep and cattle [46]. For small ruminants, there are reports of increased vocalization especially in stressful situations, such as isolation [52,53,54], and after painful procedures, such as castration [52,55] or disbudding in goats [56]. In the current study, the goats had free access to food and the company of other goats full-time, which would minimize this confounding factor, but the assessment of vocalization should be taken into consideration if UGAPS is evaluated when the goat is alone. The items “attitude—vocalizes” and “locomotion—walks or runs” had a higher frequency of occurrence in the morning (M1 and M4) and a reduction in the afternoon (M2 and M3), coinciding with the time point of *pain* and *analgesia*. It was decided to keep these two behaviors in the scale given their approval in the refinement. Although they may be a confounding factor in the assessment of pain in goats, their individual exclusion did not affect the cut-off point of indication of rescue analgesia. However, if the scale user decides to exclude both, there is the option of using a lower rescue analgesia score (≥2 instead of ≥3). Therefore, these items may or may not be evaluated at the discretion of the user of this tool. This method of excluding, according to the circumstances, one or more items when using a pain scale, has already been used in other scales, provides more versatility, and facilitates the use of the instrument [16].

As expected, the occurrence of pain-related and maintenance behaviors was, respectively, higher and lower in the postoperative periods compared to baseline, reflecting the importance of combining both types of behaviors when constructing a pain scale.

The training and selection of evaluators are relevant steps to ensure good reliability. In the present study, evaluators had previous experience in evaluating pain scales and were familiar with the scale’s behaviors after undergoing previous training, which may have favored the reliability results [29,57]. Similar results were found in the scales of other production animals, such as cattle [8], pigs [9], and sheep [10]. The intra- and inter-observer reliabilities for the total score of UGAPS were very good for all evaluators, which guarantees the repeatability and reproducibility of the UGAPS [29,57]. The limited time of daily evaluation of the videos reduces the fatigue of the evaluators, and may also have contributed to ensuring these good results [23]. Previous training must be performed by watching the videos corresponding to each behavior described for UGAPS (Table 1) or by assessing by the https://animalpain.org/en (accessed on 27 June 2023) website or Vetpain application https://play.google.com/store/apps/details?id=com.vetpain.app&pli=1 (accessed on 27 June 2023).

As one of the measures of construct validity, the multiple association evaluates the main components of the scale. A multivariate analysis lists the items in a grouped way, separating them, when similar, in the same dimension [15,16,23]. Other validated pain scales for other domestic and production animal species also used principal component analysis [8,9,10,15,16,18,21,22,23]. The UGAPS presented a unidimensional structure [30,31], as in cattle [8], sheep [10], and pigs [9], because the factor analysis did not improve when using models with two or five dimensions. However, from the biological point of view, the UGAPS includes multiple factors such as time (response to analgesia) and sensory (“attention to affected area”), motor (“posture” and “locomotion”), and emotional (“interaction”) dimensions, which biologically characterizes it as multidimensional [10,23].

Another analysis evaluated for construct validity is internal consistency [32,33,34]. The UGAPS showed Cronbach’s α as minimally acceptable [34,35]. The heterogeneity of items can reduce the α [58] and the minimally acceptable results of internal consistency may have occurred due to the variety of pain and maintenance behaviors present in the UGAPS. This result is below other scales developed for cats (0.86) [16], cattle (0.87) [8], pigs (0.82) [9], sheep (0.81) [10], and rabbits (0.78) [23], however, it is similar to that of scales for horses (0.6) [59] and donkeys (0.64) [22]. To compensate for the result of Cronbach’s α, the internal consistency of the scale reduced in 80% of the items when they were excluded, which demonstrates their contributions to the scale as a whole. The Cronbach’s α coefficient is considered the most popular method for checking internal consistency, however, this method assumes all items are, at the population stage, tau-equivalent and therefore have equal covariances. To overcome this limitation, McDonald’s total ω coefficient was applied [60]. According to this analysis, internal consistency improved to acceptable, however, in this case, only 50% of the items, when excluded, decreased total internal consistency.

The item-total correlation is also one of the analyses that evaluate construct validity, that is, scale homogeneity. Correlations between 0.3 and 0.7 are acceptable, demonstrating that the items correlated adequately with the total scale and contribute to the final version of the scale [34]. Values below 0.3 indicate that the evaluated item contributes little or little correlates with the scale, and values above 0.7 indicate that the items are repetitive or redundant because they are measuring the same attribute [34]. All UGAPS items correlated within this limit, except for the items “attention to affected area—looks at or licks” and “stamps pelvic limb”, which also showed little correlation with the other scale items by the correlogram. These items were not excluded from the scale as they had been approved in other analyses.

A tool used for measurement needs to present high sensitivity and specificity to correctly identify animals with (true positives) and without pain (true negatives), respectively, and thus to provide analgesia when necessary and not treat animals devoid of pain unnecessarily [23,34]. All items except “attitude—vocalizes” were specific, possibly due to the variation in this behavior throughout the day, even in animals devoid of pain. The scale as a whole is highly specific to correctly classify goats that do not feel pain. As for the correct classification of the goats that feel pain, although only half of the individual items showed sensitivity [34,61], the scale as a whole showed sensitivity.

Criterion validity assesses whether the instrument presents results comparable to an already established method for evaluating the same purpose [16,36]. For the UGAPS, as there is no other validated scale in the literature to assess pain in goats, it was compared with the VAS, as previously described in other species [8,9,10,15,16,18,21,22,23]. The UGAPS presented a high correlation with the VAS, which guarantees the validity of the criterion for what is feasible. Concurrent criterion validity has also been confirmed by another method which was the very good agreement between observers (reproducibility). Another step for assessing criterion validity is predictive criterion validity which was confirmed by a high percentage of goats receiving intervention analgesia because their scores were above the Youden index at the first time point after surgery (sensitivity) [61,62].

Logistic regression scaled the degree of importance of each UGAPS behavior [34]. The two most important behaviors were maintenance ones, which highlights that when these behaviors are absent there is a high probability that the animal is feeling pain. These differences in weightings indicate that the contribution of each behavior has a different importance for the need for analgesia.

The responsiveness of a scale detects differences between time points when determining how much analgesic intervention affects pain scores [15]. All items were responsive to pain, that is, scores for maintenance behaviors decreased and pain behaviors increased after surgery when compared to the baseline time point. The responsiveness of the full scale as a whole followed the proposed hypothesis, that is, the lowest score occurred at the baseline time point, there was a peak of pain after orchiectomy, followed by a significant reduction in the total score after analgesia, and a new reduction 24 h after orchiectomy, demonstrating responsiveness to different degrees of pain (severe, moderate, and mild). The same result occurred for the indication of rescue analgesia and VAS. Similar results have been described in other species [8,9,10,16,18,23]. Thus, it can be concluded that the analgesic protocol was at least partially effective, which is justified by the fact that the plasma concentration of meloxicam by the intramuscular route remains high in goats from eight to forty-eight hours after administration [63,64], although the effect of morphine is short.

The behaviors “posture—unstable” and “locomotion—standing still”, instead of being reduced, increased after rescue analgesia. A plausible explanation would be sedation caused by analgesics (morphine in this study) or sedatives, as observed in dehorned kids [2,45] and in rabbits [23]. Another possibility could be insufficient analgesia, however, the protocol used apparently has efficacy in goats [65] and was efficient in other species with previously validated scales [8,9,10,15,18,21,22,23]. The two behaviors related to the sensitive dimension of pain “attention to affected area—looks at or licks” and “unstable—stamps pelvic limb”, gradually increased over time, which is similar to a previous study in goats submitted to dehorning or castration and may be related to progressive local inflammation troubling the animal [45,46]. The “attention to affected area—looks at or licks” behavior was also relevant to identify pain in cattle [8] and lambs submitted to orchiectomy [66,67], but not in sheep after laparoscopy [10]. This discrepancy may be related to the type and location of the surgical procedure and/or the intensity of pain.

Faced with the confirmation results of the tests of the three hypotheses, the relationship between items by principal component analysis, internal consistency, and item-total correlation, and the relationship between the UGAPS and the VAS, we can confirm the construct validity of the UGAPS, as described for other pain scales in production animals [8,9,10].

The score indicating the need for analgesia was one third of the total value of the scale, in line with what was reported for other animal species [8,9,10,16,18,23]. It should be noted that although the cut-off point is a useful tool to assist in decision-making regarding rescue analgesia, clinical experience and the context in which the animal is inserted should help in this decision [10]. The high percentage of AUC demonstrated an excellent discriminatory capacity of the UGAPS, with high sensitivity and specificity. These results may be useful to improve diagnosis and, consequently, the treatment of pain in goats.

The finding that a considerable percentage of animals fell into the diagnostic uncertainty zone, increases the probability of errors in decision-making for analgesia. To avoid this limitation, the scale user may adopt the cut-off point ≥4, which is the superior limit of the diagnostic uncertainty zone, to be surer that the goat is truly suffering from pain.

The UGAPS was based on the evaluation of videos in the presence of an observer to mimic the evaluation of pain in goats in the clinical routine of a real situation. However, it is necessary to carry out this analysis in clinical situations to guarantee its reproducibility and to investigate whether, in the same way as in other species [23], the presence of an observer influences the pain scores.

Some limitations of this study should be pointed out. The UGAPS was validated only for use in young, healthy goats of two breeds, submitted to only one type of soft tissue surgery (orchiectomy), and was evaluated by evaluators with some experience. There is a need for complementary studies to test the scale with other surgical procedures, other types of clinical pain and chronic pain, other age groups, and less experienced or naive evaluators.

## 5. Conclusions

Under the conditions of this study, the UGAPS is a quick (4 min or less), simple, practical, and effective instrument to assess pain in goats by trained evaluators, as it does not require restraint and handling of the animals. The basis of the ethogram and content evaluation and the results of reliability, item-total correlation, internal consistency, specificity, sensitivity, and responsiveness guarantee content, criterion, and construct validity followed COSMIN guidelines. Additionally, the defined cut-off point for rescue analgesia (≥3 of 10) supports decision-making and selection of an analgesic protocol in animals that potentially suffer pain.

## Figures and Tables

**Table 1:** Final version (ready to use) of the Unesp-Botucatu goat acute pain scale (UGAPS) after refinement and validation (please see Appendix A for a description of behaviors).

Items	Subitems	Score	Link to Videos
Posture	It is not normal or does not jump or it is not in a bipedal position *	1	https://youtu.be/xhkj61w4DwA
	Unstable	1	https://youtu.be/1g02gI1vDo8
	Difficulty or attempting to lie down	1	https://youtu.be/9eQcGNhWGVw
Locomotion	*Does not walk or run*	1	https://youtu.be/oTSmHRysMlk
	Lying down motionless	1	https://youtu.be/PZV21z4MN4g
	Standing still	1	https://youtu.be/WpRUi1Zk2wg
Attitude	*Does not vocalize*	1	https://youtu.be/PwUdpmtI3jc
Interaction	*Does not interact with another goat (by smelling) or with the environment*	1	https://youtu.be/We3DBUdlKVQ
Attention to affected area	Looks at or licks	1	https://youtu.be/r9-7XLo_fcc
	Stamps pelvic limb	1	https://youtu.be/DNPSw_mbV24
TOTAL score		10	

* Maintenance behaviors are highlighted in *italic* and must be scored as “1” when absent. Other behaviors represent pain and must be scored as “1” when present.

**Table 2:** Item-total correlation coefficient and internal consistency of the UGAPS.

Item	Internal Consistency		Item-Total Correlation (Polychoric)
	Cronbach’s Alpha (α)	McDonald’s Omega (ω)	
	0.60	0.67	
	Excluding Each Item Below:		
*Posture: it is not normal or does not jump or it is not in a bipedal position*	**0.50**	**0.60**	**0.68**
Posture: unstable	**0.59**	0.68	**0.32**
Posture: difficulty lying down	**0.59**	0.69	**0.49**
*Locomotion: does not walk or run*	**0.49**	**0.59**	**0.67**
Locomotion: lying down motionless	**0.57**	**0.64**	**0.44**
Locomotion: standing still	**0.57**	0.68	**0.36**
*Attitude: does not vocalize*	**0.57**	**0.65**	**0.35**
*Interaction: does not interact*	**0.52**	**0.62**	**0.53**
Attention to affected area: looks at or licks	0.63	0.69	−0.04
Attention to affected area: stamps pelvic limb	0.64	0.69	−0.12

UGAPS: Interpretation of item-total correlation: accepted items >0.3 and <0.7 (bold). Interpretation for α: 0.60–0.64, minimally acceptable; 0.65–0.69 acceptable; 0.70–0.74 good; 0.75–0.80 very good, and >0.80 excellent [33,34]. Interpretation for the ω: 0.65–0.80 acceptable; >0.80 strong [35]; values of α and ω that are lower than the values found for the total set of items are highlighted in bold.

**Table 3:** Specificity and sensitivity in percentages of each item of the UGAPS.

Item	Specificity (%)	Sensitivity (%)
*Posture: it is not normal or does not jump or it is not in a bipedal position*	**99 (96–100)**	**78 (72–83)**
Posture: unstable	**98 (87–100)**	55 (50–61)
Posture: difficulty lying down	**100 (87–100)**	53 (48–59)
*Locomotion: does not walk or run*	**99 (96–100)**	**76 (70–81)**
Locomotion: lying down motionless	**99 (95–100)**	**71 (66–76)**
Locomotion: standing still	**96 (88–99)**	60 (54–65)
*Attitude: does not vocalize*	62 (56–68)	**78 (69–84)**
*Interaction: does not interact*	**86 (81–91)**	**82 (77–87)**
Attention to affected area: looks at or licks	**81 (54–96)**	51 (46–56)
Attention to affected area: stamps pelvic limb	**70 (35–93)**	50 (45–56)
UGAPS total score	**99 (96–100)**	**90 (86–94)**
Indication of analgesia	**100 (98–100)**	**93 (89–96)**

UGAPS: Interpretation of specificity and sensitivity: excellent 95–100%; good 85–94.9%; moderate 70–84.9%; non-specific or sensitive <70%; values in bold >70%. Maintenance behaviors are written in italics [34].

**Table 4:** Responsiveness of each item and total score of the UGAPS, VAS, and rescue analgesia indication (median (range)).

ITEMS	Baseline M1	Pain M2	Analgesia M3	24 h after M4
Posture: it is not normal or does not jump or it is not in a bipedal position *	0 (0–0) ^d^	1 (0–1) ^a^	0 (0–1) ^b^	0 (0–0) ^c^
Posture: unstable	0 (0–0) ^c^	0 (0–0) ^b^	0 (0–1) ^a^	0 (0–0) ^b^
Posture: difficulty lying down	0 (0–0) ^d^	0 (0–0) ^a^	0 (0–0) ^c^	0 (0–0) ^b^
Locomotion: does not walk or run *	0 (0–0) ^c^	1 (0–1) ^a^	1 (0–1) ^a^	0 (0–0) ^b^
Locomotion: lying down motionless	0 (0–0) ^c^	1 (0–1) ^a^	0 (0–0) ^b^	0 (0–0) ^b^
Locomotion: standing still	0 (0–0) ^c^	0 (0–1) ^b^	1 (0–1) ^a^	0 (0–1) ^b^
Attitude: does not vocalize *	1 (0–1) ^d^	1 (1–1) ^b^	1 (1–1) ^a^	1 (0–1) ^c^
Interaction: does not interact *	0 (0–0) ^d^	1 (1–1) ^a^	1 (0–1) ^b^	0 (0–1) ^c^
Attention to affected area: looks at or licks	0 (0–0) ^c^	0 (0–0) ^bc^	0 (0–0) ^b^	0 (0–1) ^a^
Attention to affected area: stamps pelvic limb	0 (0–0) ^c^	0 (0–0) ^c^	0 (0–0) ^b^	0 (0–1) ^a^
Rescue analgesia	0 (0–0) ^d^	1 (1–1) ^a^	1 (1–1) ^b^	0 (0–1) ^c^
VAS (cm)	0.42 ± 0.05 ^d^	6.73 ± 0.15 ^a^	5.54 ± 0.17 ^b^	3.44 ± 0.18 ^c^
Total UGAPS score (mean ± SD)	0.73 ± 0.69 ^d^	4.46 ± 1.3 ^a^	4.09 ± 1.38 ^b^	2.88 ± 1.51 ^c^
Total UGAPS score (median (min-max))	1 (0–3) ^d^	5 (0–7) ^a^	4 (1–7) ^b^	3 (0–7) ^c^

* Maintenance behaviors (italic). Rescue analgesia (0—no indication; 1—indication); VAS (0–10). Different letters express significant differences between time points where a > b > c > d. Pain-related behaviors 0—absent and 1—present; maintenance behaviors 0—present and 1—absent.

**Table 5:** Youden index, optimal cut-off point for indication of analgesia, specificity, sensitivity, and AUC corresponding to an indication for rescue analgesia of the UGAPS and VAS.

Parameters	UGAPS (Total Sum)	UGAPS (Excluding Vocalizes)	UGAPS (Excluding Walks/Runs)	UGAPS (Excluding Vocalizes and Walks/Runs)	VAS
Optimal cut-off	2.5 (2.5–3.5)	2.5 (1.5–2.5)	2.5 (2.5–2.5)	1.5 (1.5–1.5)	3.35 (3.2–3.75)
Specificity	86 (83–95)	93 (85–96)	88 (84–90)	84 (80–88)	96 (94–99)
Sensitivity	94 (84–96)	87 (84–94)	90 (87–93)	93 (91–96)	98 (95–100)
AUC	95 (94–97)	95 (94–97)	94 (93–94)	94 (93–96)	91 (98–100)
UGAPS AUC vs. AUC without the behaviors and VAS		0.9809	0.0028	0.0285	5.922^−08^

## Data Availability

The data presented in this study are available in the Supplementary Materials according to “MDPI Research Data Policies” at https://www.mdpi.com/ethics (accessed on 27 June 2023).

## Supplementary Materials

The following supporting information can be downloaded at: https://www.mdpi.com/article/10.3390/ani13132136/s1, Figure S1: (A) Goats housed in the pen. The red arrows indicate the locations of the feeding troughs, and the yellow arrows indicate the location of the two automatic drinking troughs. (B) Identification of goats with numbers painted on the right and left flanks of the animals (animal numbers 2 and 3). The yellow arrows were inserted in the footage to help identify the animal to be evaluated in each video (in this example, goat number 3). In the upper left corner of the image, the presence of the observer (MWF) can be seen, who was always the same individual, positioned in the same place, in all the footage. Figure S2. Correlogram and heat map (polychoric correlation coefficient) between items on the UGAPS. Caption: Lighter colors demonstrate a greater correlation between behaviors. Figure S3. Weighting of behaviors based on the UGAPS binomial multilevel logistic regression model.

## Appendix A. Ethogram of Goats Submitted to Orchiectomy

**No.**	**Behavior**	**Definition**
**1**	**Walks or runs**	Explores the pen by walking or running.
**2**	**Lying down motionless**	Lying down with abdomen and chest in contact with the floor, with limbs tucked under the body.
**3**	**Standing still**	In a quadrupedal position, without moving and with the limbs in contact with the ground, normally oblivious to the environment.
**4**	**Bipedal position**	Supports the body on the pelvic limbs while the forelimbs are supported on the pen (climbing).
**5**	**Normal position**	In quadrupedal posture, with the limbs in contact with the ground. Attentive to the environment.
6	Jumps	Jumps around the bay.
**7**	**Interacts with the bay**	Interacts with the stall by nibbling or smelling.
8	Eats	Ingests food from the trough.
**9**	**Interacts with trough**	Interacts with the trough inside the stall (bites, digs, pulls, and/or pushes).
10	Headbutts	Pushes or hits another goat with its head.
**11**	**Interacts by smelling**	Smells another goat’s anus, penis, muzzle, or any other body part.
12	Wags the tail	Wags tail slowly or quickly, with hind limbs on the ground.
13	Arches back	Contracts the lumbar region dorsally.
14	Tremors	Presents tremors of the head, ear, and trunk.
**15**	**Looks at and/or licks the wound**	Moves the head towards the flank looking back or licks the inguinal region (wound).
16	Difficulty or attempts to get up	Tries to get up but stays lying down.
**17**	**Difficulty or attempts to lie down**	Attempts to lie down, lies down with difficulty, or remains standing up.
**18**	**Stamps pelvic limb** **(may wag tail)**	Wags tail quickly and vigorously while stamping a hind limb on the ground.
19	Stretches pelvic and/or thoracic limb	Stretches the thoracic and/or pelvic limb while lying down.
20	Rests head on the ground	Lying down with the muzzle resting on the floor of the stall.
**21**	**Unstable posture**	Loses balance or wobbles in quadrupedal position.
22	Interacts with tire	Interacts with enrichment (tire).
23	Digs	Digs the stall floor with forelimbs.
24	Drinks water	Drinks water from the troughs.
25	Self-cleaning	Scratches itself with pelvic limbs or nibbles.
26	Ruminates	Ruminates in quadrupedal or lying position.
27	Sneezes/coughs	Sneezes.
28	Yawns	Opens its mouth.
29	Urinates	Opens pelvic limbs and urinates.
30	Defecates	Defecates with the tail upright.
31	Looks out from the bay	Keeps the eyes out of the window, attentive to the external environment.
32	Licks/sniffs penis	Licks or sniffs own penis.
33	Stumbles	Animal stumbles on the floor of the stall, trough, or tire.
**34**	**Vocalizes**	Calls out with an open mouth.
35	Settles down	Lies down with difficulty finding a position.
36	Stretches out	Stretches the limbs and body.
37	Flehmen	Performs the Flehmen response by contracting the upper lip.
38	Mounts	Mounts another goat.
39	Attentive to the observer	Looks at or interacts with the observer.
40	Ejaculates	Expels penile secretion in repetitive movements, after mounting
Behaviors in bold and highlighted were approved for the final UGAPS scale.		

## Appendix B. Unesp-Botucatu Goat Acute Pain Scale (UGAPS), Pre-Refinement, Used for Analysis by the Evaluators

(1) Posture	(select more than one option if applicable)	
(a) Normal, jumps, or bipedal position		0
(b) Unstable (loses balance or wobbles in quadrupedal position)		1
(c) Difficulty or attempts to get up		1
(d) Difficulty or attempts to lie down		1
(e) When lying down, constantly changes position		1
(f) None of the above		0
(2) Locomotion	(select more than one option if applicable)	
(a) Walks or runs		0
(b) Lying down motionless		1
(c) Standing still		1
(3) Appetite		
Present		0
Absent		1
(4) Attitudes		
(a) Digs		0
(b) Vocalizes		0
(c) Practices grooming (self-cleaning, except in the affected region) with mouth/pelvic limbs		0
(d) Tremors		1
(e) None of the above		1
(5) Interaction with the environment		
Interacts (comes into contact with the environment or smells or nibbles or plays) with the environment (tire, trough, pen)		0
Does not smell or interact with the environment		1
(6) Interaction with another goat		
(a) Interacts by smelling		0
(b) Interacts by headbutting		0
(c) Interacts by mounting		0
(d) Does not interact		1
(7) Pays attention to affected area	(select more than one option if applicable)	
(a) Looks at or licks		1
(b) When in recumbency stretches the pelvic limbs		1
(c) Stamps pelvic limb on the ground (may wag tail)		1
(d) Arches back		1
(e) None of the above		0
