# Supplementary material for: Development and Validation of the Unesp-Botucatu Goat Acute Pain Scale
Source: Animals (Basel). 2023 Jun 28;13(13):2136. doi: 10.3390/ani13132136 (PMC10339886; doi:10.3390/ani13132136)
Supplement: Supplementary file 1 [file animals-13-02136-s001.zip › animals-2442092-supplementary.pdf]

Supplementary material

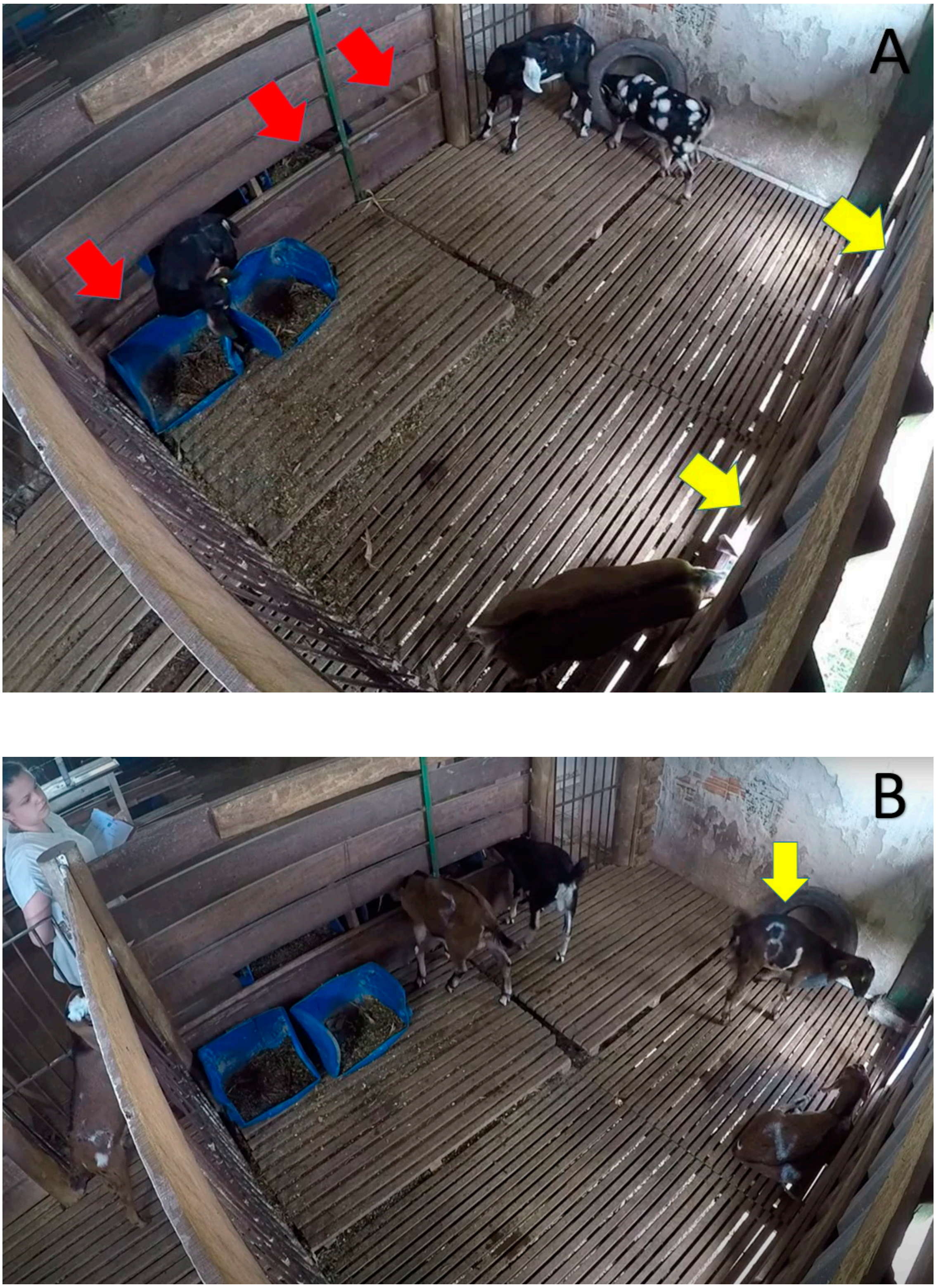

Figure S1 (A) - Goats housed in the pen. The red arrows indicate the locations of the feeding troughs, and the yellow arrows indicate the location of the two automatic drinking troughs. **(B)** - Identification of goats with numbers painted on the right and left flanks of the animals (animal numbers 2 and 3). The yellow arrows were inserted in the footage to help

identify the animal to be evaluated in each video (in this example, goat number 3). In the upper left corner of the image, the presence of the observer (MWF) can be seen, who was always the same individual, positioned in the same place, in all the footage.

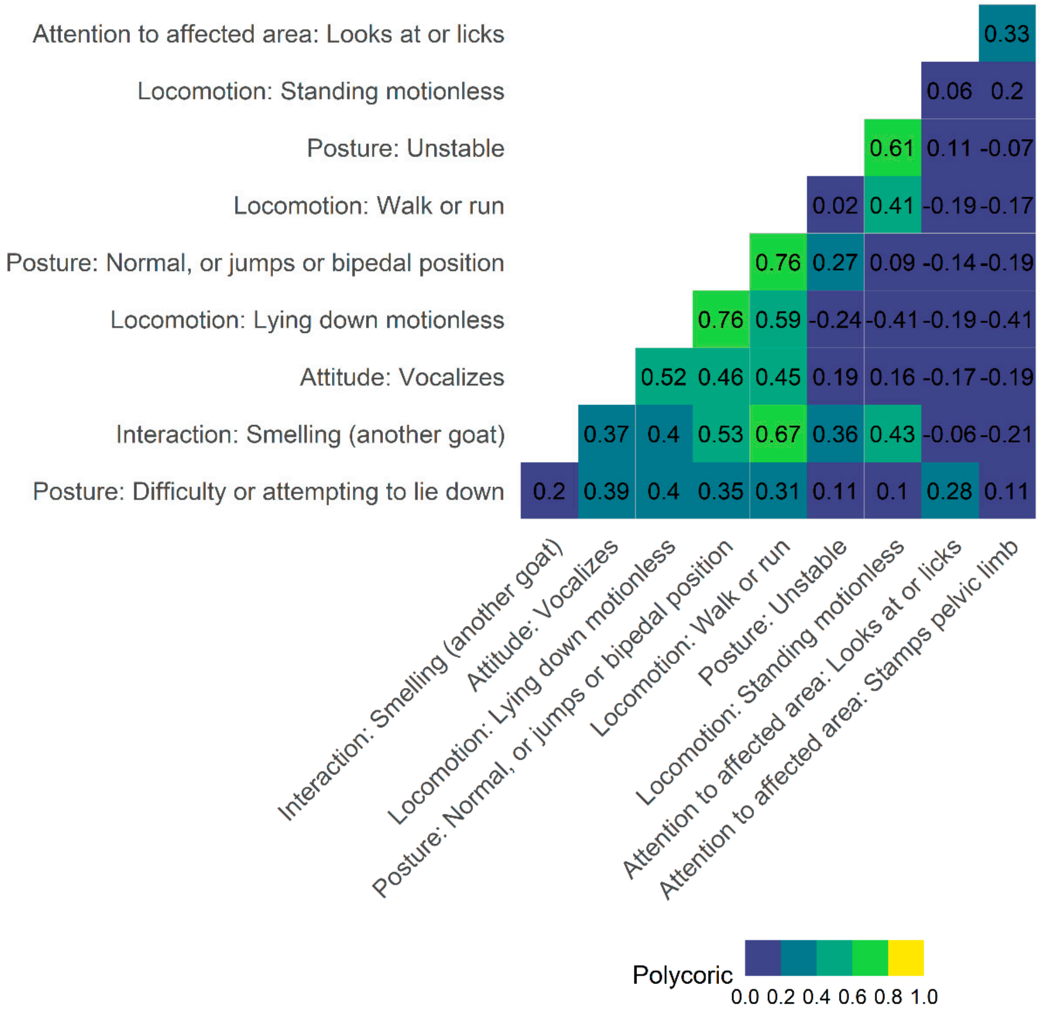

Figure S2. Correlogram and heat map (polychoric correlation coefficient) between items on the UGAPS. Caption: Lighter colors demonstrate a greater correlation between behaviors.

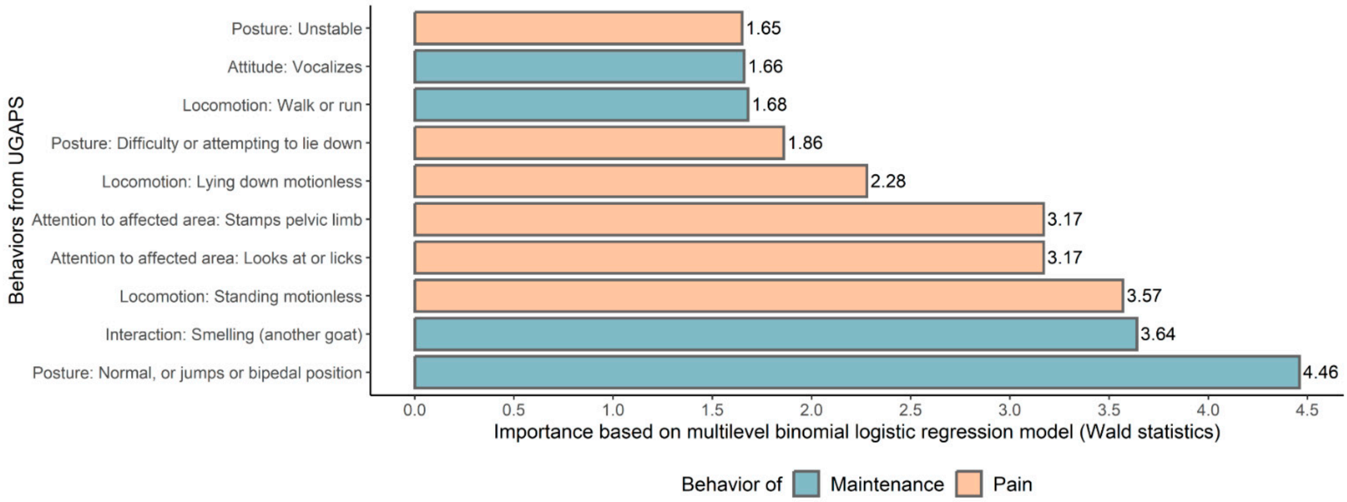

Figure S3. Weighting of behaviors based on the UGAPS binomial multilevel logistic regression model.
